# Supplementary material for: Deletion of KLF10 Leads to Stress-Induced Liver Fibrosis upon High Sucrose Feeding
Source: Int J Mol Sci. 2020 Dec 30;22(1):331. doi: 10.3390/ijms22010331 (PMC7794950; doi:10.3390/ijms22010331)
Supplement: Supplementary file 1 [file ijms-22-00331-s001.pdf]

Supplementary data to International Journal of Molecular Sciences

Deletion of KLF10 Leads to Stress-Induced Liver Fibrosis upon High Sucrose Feeding

Junghoon Lee[1,2], Ah-Reum Oh[1,2], Hui-Young Lee[1,2], Young-Ah Moon[3], Ho-Jae Lee[1,2,*], and Ji-Young Cha[1,2,4,*]

[1] Department of Biochemistry, Lee Gil Ya Cancer and Diabetes Institute, Gachon University College of Medicine, Incheon 21999, Korea; hun21321@naver.com (J.L.); areum1030@naver.com (A.-R.O.); hylee@gachon.ac.kr (H.-Y.L.)

[2] Department of Health Sciences and Technology, GAIHST, Gachon University, Incheon 21999, Korea

[3] Department of Molecular Medicine, Inha University School of Medicine, Incheon 22212, Korea; yamoon15@inha.ac.kr (Y.-A.M.)

[4] Gachon Medical Research Institute, Gachon University Gil Medical Center, Incheon 21565, Korea

* Correspondence: hojlee@gachon.ac.kr (H.-J.L.); jycha1014@gachon.ac.kr (J.-Y.C.); Tel.: +82-32-899-6054 (H.-J.L.); +82-32-899-6070 (J.-Y.C.)

Supplementary table S1. Pathological scores for HSD-induced NAFLD.

Supplementary table S2. Primer sequences for quantitative real-time PCR.

Supplementary table S1. Pathological scores for HSD-induced NAFLD.

**Steatosis**	**definition**	**Score**
0-3	Grade Low- to medium-power evaluation of parenchymal involvement	
	<5%	0
	5-33%	1
	33%-66%	2
	>66%	3
	Predominant distribution pattern	
	Zone3	0
	Zone1	1
	Azpma;	2
	Panacinar	3
**Fibrosis**		
0-4	None	0
	Perisinusoidal or periportal	1
	Mild, zone 3, perisinusoidal	1.2
	Moderate, zone 3, perisinusoidal	1.4
	Portal/periportal	1.6
	Perisinusoidal and portal/periportal	2
	Bridging fibrosis	3
	Cirrhosis	4
Inflammation		
	Lobular inflammation Overall assessment of all inflammatory foci	
	No foci	0
	<2 foci per 200 field	1
	2-4 foci per 200 field	2
	>4 foci per 200 field	3
	Microgranulomas Small aggregates of macrophages	
	Absent	0
	Present	1
	Large lipogranulomas Usually in portal areas or adjacent to central veins	
	Absent	0
	Present	1
	Portal inflammation Assessed from low magnification	
	None to minimal	0
	Greater than minimal	1
Cell injury		
	Ballooning*	
	None	0
	Few balloon cells	1
	Many cells/prominent ballooning	2
	Acidophil bodies	
	None to rare	0
	Many	1
	Pigmented macrophages	
	None to rare	0
	Many	1
	Megamitochondria*	
	None to rare	0
	Many	1

Supplementary table S2. Primer sequences for quantitative real-time PCR

Gene symbol	Forward primers (5' to 3')	Reverse primers (5' to 3')
Apob	CGTGGGCTCCAGCATTCTA	TCACCAGTCATTTCTGCCTTTG
Atf3	TGGAGATGTCAGTCACCAAGTCT'	GCAGCAGCAATTTTATTTCTTTCT"
Catalase	CCGAGTCTCTCCATCAGGTTT	TCATGTGCCGGTGACCAT
Cd36	GGAACTGTGGGCTCATTGC	CATGAGAATGCCTCCAAACAC
Chop	CACCACACCTGAAAGCAGAAC	GGTGAAAGGCAGGGACTCA
Col1a1	CCTGAGTCAGCAGATTGAGAACA	CCAGTACTCTCCGCTCTTCCA
Fas	GCTGCGGAAACTTCAGGAAAT	AGAGACGTGTCACTCCTGGACTT
Gadd34	GGCGGCTCAGATTGTTCAA	GAAGTGTACCTTCCGAGCTTTTAGAG
Mcp1	TCTGGGCCTGCTGTTCACA	CCAGCCTACTCATTGGGATCA
Mttp	AGCCAGTGGGCATAGAAAATC	GGTCACTTTACAATCCCCAGA
Noxa	TGGGAAGTCGCAAAAGAGCA	CCCTGCTCAGGGAACCAAAA
Nqo1	CCATGGCGGCGAGAAG	TTCATGGCGTAGTTGAATGATGT
Nrf2	CCCGGTTGCCCACATTC	TGTCTCTGCCAAAAGCTGCAT
Pai1	GCAAAAGGTCAGGATCGAGGTA	GTGCCGAACCACAAAGAGAAAG
Puma	GCGGCGGAGACAAGAAGA	AGTCCCATGAAGAGATTGTACATGAC
Smad3	GTAATGCACTTGGTGTTCACGTT	TGAAGAAGCTCAAGAAGACGGG
Sod2	CAGACCTGCCTTACGACTATGG	CTCGGTGGCGTTGAGATTGTT
Srxn1	CACAACGTACCAATCGC	CCTTTGATCCAGAGGACGTC
Tgfb1	GCAGTGGCTGAACCAAGGA	AGAGCAGTGAGCGCTGAATC
Tnfα	CTGAGGTCAATCTGCCCAAGTAC	CTTCACAGAGCAATGACTCCAAAG
α-Sma	CACGGCATCATCACCAACTG	GGCCACACGAAGCTCGTTAT
